# Trends in physician diagnosed gout and gout therapies in the US: results from the national ambulatory health care surveys 1993 to 2009

**DOI:** 10.1186/ar4370

**Published:** 2013-11-06

**Authors:** Eswar Krishnan, Linjun Chen

**Affiliations:** 1 Department of Medicine, Stanford University, ARAMIS Program, Stanford, CA, 94305, USA

## Abstract

**Introduction:**

Gouty arthritis (gout) is primarily cared for in ambulatory care settings. Although the prevalence of gout in the US is thought to be increasing, there have been few data on this as well as temporal changes in gout medication use.

**Methods:**

We analyzed annual visit and drug utilization data from national sample surveys of physician practices and hospital outpatient clinics in the US from 1993 to 2009. Gout diagnosis was recorded by individual physicians.

**Result:**

The frequency of visits for gout increased three-fold from 1993 through 2009; most of the increases were observed from 2003 onwards. The increase was only partly explained by changes in age and gender composition of the surveys over time. A concomitant increase in prescriptions for allopurinol and colchicine and decrease in prescriptions for anti-inflammatories was observed. Aspirin use, a putative risk factor for gout and gout flares, increased substantially over this period. Probenecid use was negligible. Frequency of systemic steroid use has not changed over time.

**Conclusions:**

The number of ambulatory visits for gout has increased almost three-fold in the first decade of the millennium coinciding with increases in physician and patient awareness. This increase was primarily due to visits among the elderly. Uricosuric use remained negligible whereas the uses of allopurinol and colchicine have increased rapidly. Use of traditional non-steroidals has declined, possibly due to safety concerns whereas glucocorticoid use remains unchanged.

## Introduction

Gout is a common type of arthritis that is mostly managed in ambulatory (outpatient) care settings. Some reports suggest that its prevalence may be increasing [1,2]. Over the past 20 years, therapies for gout have undergone some changes including the introduction and subsequent withdrawal of Cox-1-sparing anti-inflammatory therapies, increasing recognition of cardio-metabolic impact of gout, and the poor health-related quality of life associated with gout [3-8]. Cross-sectional studies and short-term follow-up studies have suggested that uncontrolled gouty arthritis (gout) is associated with significant health care utilization and costs in ambulatory care; this is especially true among those with uncontrolled gout [5,9,10]. However there have been few prospective studies that have addressed longer-term trends in ambulatory care use and prescription of treatments for gout.

There were two goals for the present study. The first was to assess the frequency of gout-related ambulatory care visits in the US from 1993 through 2009. The second was to describe the time trends in prescriptions for urate-lowering treatment and anti-inflammatory therapy for gout.

## Methods

### Data source and data description

This study used publicly available de-identified data, and as per the Stanford institutional review board does not constitute Human Subject Research, nor does it need individual participant consent.

Ambulatory care in the US is provided through two distinct settings - hospital based clinics and non-hospital based clinics. The latter includes freestanding private practices (physician offices) as well as health system outpatient clinics. We used 17-year data from the National Ambulatory Medical Care surveys (NAMCS) and National Hospital Ambulatory Medical Care Surveys (NHAMCS) from 1993 through 2009 to estimate the number of medications. These two nationally representative, annual, sister-surveys, conducted by the National Center for Health Statistics collect demographic, diagnosis and prescription data from a national sample of ambulatory care visits to physician offices (NAMCS) and hospital-based clinics (NHAMCS). Both NAMCS and NHAMCS use multi-stage probability sampling design to obtain a sample of visits that are representative of all outpatient visits in the US for a given year. NAMCS data sampling has three stages. In the first stage, primary sampling units (PSUs) are selected from geographically defined areas. In the second stage, a probability sample of practicing physicians is selected within each PSU. In the final stage patient visits are selected within the annual practices of sampling physicians. NHAMCS has similar sampling design to NAMCS with the same first stage and the same last stage, but it has two intermediate stages instead of one. Hospitals within PSUs are sampled in the second stage and then clinics within those hospital outpatient departments are sampled further in the third stage. These two care settings can be combined within years because they have different sampling frames. The data elements used in the present study have been collected without changes over the study period from 1993 to 2009. So data for 1993 to 2009 from the two surveys have the same structure and hence they could be combined for the data analysis. Extensive description of the survey and methodology are available in the public domain [11] (accessed 25 September 2012).

The NAMCS and NHAMCS data are mostly uniformly formatted for each year. For each record, besides the design variables (PSU, Stratum and Weight) we have the information as to whether or not the patient has gout at that visit, and whether or not he/she takes gout-related medications, such as colchicine, steroid, etcetera, for gout.

#### Diagnoses

For each visit, up to three diagnoses directly related to the visit, as determined by the physician, were coded using International Classification of Diseases 9^th^ revision, Clinical Modification codes. Thus, a visit exclusively for sinusitis will be coded as having one diagnosis (sinusitis) even if the patient had numerous other diagnoses such as cancer, compression fractures, etcetera.

#### Prescription medications

Up to six medications either prescribed or continued during the visit were coded and recorded using National Drug Code Directory (prior to 2006) and Lexicon Plus® starting in 2006 [12] (accessed 20 September 2012). Dosage information was not available. Anti-inflammatory therapies were classified as followed: aspirin, non-aspirin anti-inflammatories (including coxibs), systemic corticosteroids (prednisone, prednisolone, methyl prednisolone) and colchicine.

#### Case definition of gout

In the NAMCS and NHAMCS datasets, a diagnosis of gout can be inferred based on two data items, a physician-assigned visit diagnosis, and a prescription of gout medications. A number of patient visits were not associated with recorded-visit diagnosis of gout but received a prescription of allopurinol or colchicine (typically refills), and vice versa. For the purpose of this study, a patient was determined to have gout if it was associated with a recorded visit diagnosis of ICD-9 CM code 274.*, and/or had a prescription of allopurinol or colchicine. Additional clinical information about gout severity and chronicity were not available.

#### Inclusion and exclusion

We excluded all patients with age <20 or >90 years for analyses that involved gout, because gout is very rare in the younger category, and the number of observations in the >90 years category were too few.

### Statistical analyses and interpretation

#### Data characteristics

There are two special aspects of NAMCS and NHAMCS datasets that have a bearing on the statistical analyses. The first is the complex survey design that mandated incorporation of design factors and survey weights, for overall analyses and analyses within the subsets (domains). This was accomplished using SVY module of the software STATA 12.1® (Statacorp College Station, TX, USA). NAMCS and NHAMCS are surveys intended to obtain a snapshot of ambulatory care utilization and not population prevalence of disease, such as the NHANES (National Health and Nutrition Examination Survey). The second unique aspect about these data is that the sampling was intended to obtain a sample of visits and not patients. While this sample may be similar to the sample of all patients in the ambulatory clinics, it is not necessarily designed to be so. Patients with gout, who happen to visit the clinic more than once in the enrollment period, will be counted as two independent visits. Conditions with higher health-care utilization are likely to be represented disproportionate to their true prevalence in the population, whereas those of low utilization and short natural history will be under-represented. The estimates of the number of visits and prescriptions were considered reliable only if the relative standard errors (standard error of the estimate/estimate) was ≤30%.

#### Proportions, counts and rates

For the present analyses we calculated proportions and rates to assess the relative magnitudes. The numerators and denominators of the estimated proportions presented here are the numbers of visits unless specified otherwise. The estimated count of the visits for gout and the count of the number of prescriptions of medications were calculated by applying survey weights. Counts were rounded off to their nearest thousands or millions as appropriate.

#### Trend analyses

The bivariate changes in counts, proportions and rates over time were assessed visually as well formally. Trend curves were graphically fitted using polynomial regression, as they provided better fits to the non-linear data. Calendar year was treated as a continuous measure for trend testing. Wherever relevant the years were collapsed to 4- to 5-year categories.

The impact of changes in age and gender profile on the observed trends was accounted for by log-binomial implementation of generalized linear models (proportions), specifying survey weights and sampling units. The magnitude of trends was summarized by relative risk estimates/odds ratios.

## Results

### Description of the dataset

The dataset, prior to any exclusions, included 1.01 million ambulatory-care visit records from 1993 to 2009. We excluded 233,000 visits for patients age <20 years and 4,734 visits for patients age > 90 years, leaving 782,000 visits eligible for inclusion by age criterion. Of these, 4,683 patients/visits met the case definition of gout. Within the 4,683 visits in the gout group, there were 3,119 visits where allopurinol was prescribed and 54 with probenecid prescriptions. There were 854 prescriptions of non-aspirin, non-coxib anti-inflammatories, 732 prescriptions for aspirin, 115 prescriptions for coxib, and 839 prescriptions for colchicine.

### Estimated ambulatory visits for gout in the year 2009

In the year 2009 there were 12.1 (95% confidence interval (9.0, 15.2)) million ambulatory care visits for gout out of a nationwide estimated 900 (750, 1100) million visits in the 20- to 90-years age category. Of these 8.9 (6.6, 11.2) million were men and 3.2 million (2.2, 4.3) were women. There were 8.7 million (6.3, 11.2) prescriptions for allopurinol, 2.3 million (1.4, 3.1) prescriptions for colchicine, 1.7 (1.0, 2.4) million prescriptions for non-aspirin non-coxib non-steroidal anti-inflammatory drugs (NSAIDS), 409,000 (80,000, 736,000) prescriptions for coxibs 2.7 (1.8, 3.6) million prescriptions for aspirin, and 488,000 (182,000, 794,000) prescriptions for steroids.

### Time trends

#### Overall number of ambulatory visits and visits for specific causes

**Figure 1:**
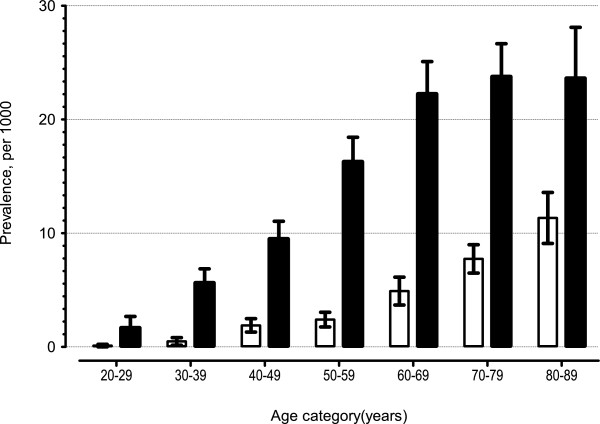
**Age-sex distribution of gout in ambulatory care settings in the US, National Ambulatory Health Care surveys, 1993 to 2009.** Prevalence was calculated as a proportion of all the ambulatory care visits in hospital-based clinics as well as physician offices in the US.

**Figure 2:**
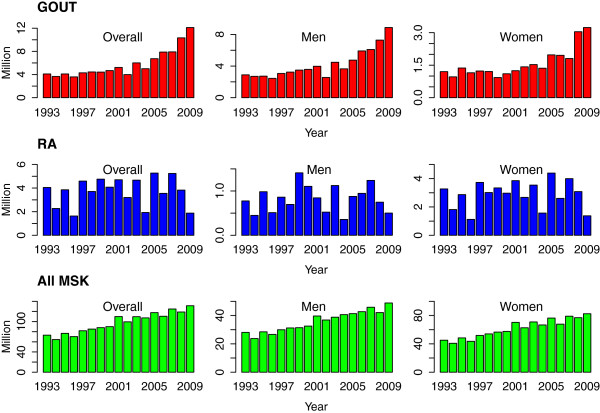
Time trends in gout visits contrasted with those for all musculoskeletal diagnoses (ICD9-CM 710.* to 739.*) and with those for rheumatoid arthritis (ICD9-CM 714.*). RA, rheumatoid arthritis; MSK, musculoskeletal diagnoses.

From 1993 to 2009 in the US, the estimated number of ambulatory care visits (for any reason) in the age category 20 to 90 years increased from 600 (520, 680) million to 900 (750, 1100) million visits. Overall, the prevalence of visits for gout, as a proportion of all visits recorded increased with age among men and women (Figure 1). Visits for musculoskeletal diagnoses (ICD9-CM 710.*-739.*) in general showed an increasing trend but the trend preceded the rise of visits for gout and appears to be a part a secular trend (Figure 2). When visits for rheumatoid arthritis (ICD9-CM 714.*) were examined, no time trends were observed.

#### Age and gender

Over the period of observation the mean age of patients seeking outpatient care in the US increased from 51 years (51, 52) to 55 years (54, 55). Among those who met our case definition of gout, the mean age was essentially unchanged: 65 (61, 69) versus 65 (64, 67). Overall, the proportion of men in the dataset was unchanged over time at approximately 38%. Among those with gout the proportion of men in 1993 was 71% (58%, 81%) and in 2009 was 73% (68%, 79%).

#### Gout

**Figure 3:**
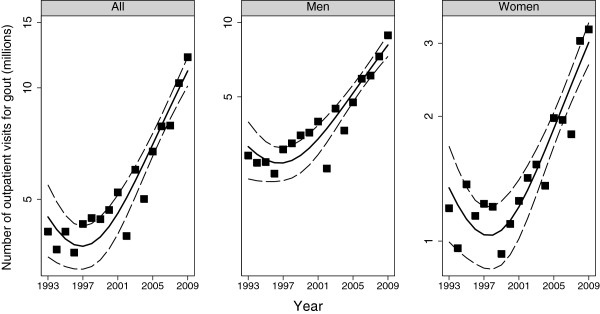
Time trends in the estimated number of ambulatory care visits for gout in the US: data from the National Ambulatory Health Care Surveys 1993 to 2009.

**Figure 4:**
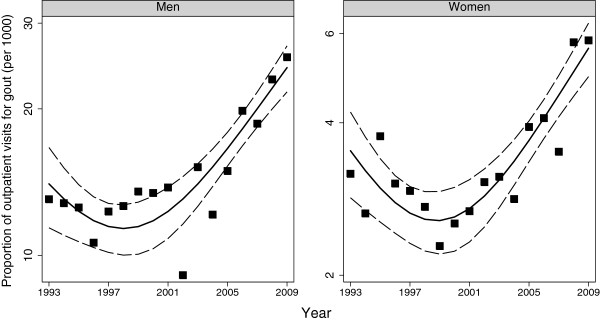
**Trends for gout as a proportion of all ambulatory visits in the US: data from the National Ambulatory Health Care Surveys 1993 to 2009.** Trend lines and 95% confidence bands were fitted using fractional polynomial regression. Gout was defined as a physician diagnosis of gout or prescription of allopurinol/colchicine.

The estimated number of visits with gout in 1993 was 4.1 (2.9, 5.3) million and this rose to 12.1 (9.0, 15.2) million in 2009 (*P* <0.001). This increase was observed in men and women (Figures 2 and 3). When examined by age, the increases were statistically significant for age >60 years among men, and among women in the 60- to 79-year age category (Table 1). Gout as a proportion of all visits decreased between 1993 and 2000 among women but subsequently showed an increase in both men and women (Figure 4). Compared to 1993, there were no statistically significant increases in the proportion among women but the proportion increased among men from 13 per 1,000 (10, 17) in 1993 to 26 per 1,000 (21, 31) in 2009. The trends were unchanged when data were reanalyzed after redefining gout exclusively based on the ICD code.

**Table 1:** Estimated number of gout visits in the US from 1993 to 2009 by age and gender

		**Men**					**Women**			
**Age, years**	**1993 to 1996**	**1997 to 2000**	**2001 to 2005**	**2006 to 2009**	***P*-value**^**a**^	**1993 to 1996**	**1997 to 2000**	**2001 to 2005**	**2006 to 2009**	***P*-value**^**a**^
20 to 39	704,285	1,039,594	769,990	1,591,411	0.09	136,926	199,082	105,708	280,295	0.62
40 to 59	3,658,756	4,903,678	5,746,758	7,944,502	0.07	1,180,143	965,395	2,037,772	1,409,484	0.81
60 to 79	5,590,948	5,836,312	10,280,899	14,921,770	<0.001	2,192,226	2,456,175	3,604,061	5,349,247	0.01
> = 80	800,007	1,585,809	2,617,818	3,721,661	0.006	1,172,668	851,594	1,784,431	3,005,855	0.06
Overall	10,753,996	13,365,393	19,415,465	28,179,344		4,681,963	4,472,246	7,531,972	10,044,881	

^a^*P*-values are for trend: the test was performed using bivariate logistic regression models with calendar year as a continuous variable.

In bivariate logistic regressions models each advancing year was associated with an odds ratio of 1.05 (1.03, 1.06). In multivariable logistic regression models that included age, sex and year, the corresponding odds ratio was 1.04 (1.03, 1.06). When the year variable was categorized as in Table 1, the period 2006 to 2009 was associated with an odds ratio of 1.8 (1.5, 2.0) in unadjusted regression and an odds ratio of 1.6 (1.4, 1.9) in age- and sex-adjusted logistic regressions.

#### Medications prescribed

**Figure 5:**
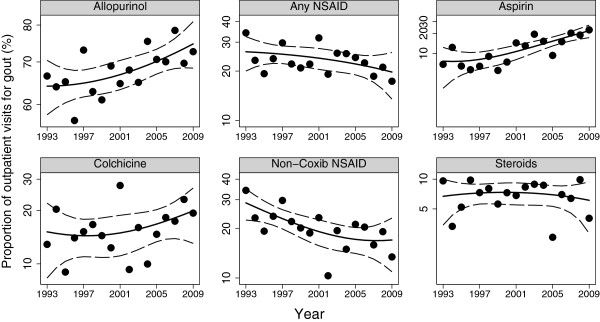
**Time trends in medications prescribed for gout in the US: data from the National Ambulatory Health Care Surveys 1993 to 2009.** Trend lines and 95% confidence bands were fitted using fractional polynomial regression. Gout was defined as a physician diagnosis of gout or prescription of allopurinol/colchicine. NSAID, non-steroidal anti-inflammatory drugs.

Figure 5 shows the proportion of visits for gout where each medication was prescribed among those who met the case definition for gout. The trends for colchicine and steroids were not statistically significant. In logistic regressions that were adjusted for age and sex, each advancing year was associated with a significant increase in the use of allopurinol with an odds ratio of 1.02 (1.00, 1.05; *P* = 0.048) and the use of aspirin with an odds ratio of 1.09 (1.05, 1.12). The use of non-coxib NSAIDs decreased with a yearly odds ratio of 0.96 (0.94, 0.99). There were no significant trends in other medications.

### Combination treatment in gout

The estimate of visits with concurrent prescriptions of allopurinol and probenecid was negligible, with approximately 50,000 over the observation period. These estimates were based on a tiny number of observations and thus, unreliable (relative standard errors >30%). The probenecid data were not analyzed further.

Overall, the combination of colchicine and any one of the NSAIDS were prescribed in 3.5 million (2.4 to 4.6 million) gout patients, representing 3.6% (2.6%, 4.8%) of all gout visits. The confidence intervals of these estimates were conservative as they overlap, whereas the *P*-value for the difference in proportion was statistically significant at 0.01. On further evaluation this was determined to be due to the conservativeness (biasing towards the null) of the standard error estimate compared to hypothesis testing and *P*-values [13].

The proportion of such combinations increased from 2.02 (1.2, 3.3) in the 1993 to 2000 period, to 4.3 (3.1, 6.1) in the 2001 to 2009 period. In unadjusted logistic regression this change represented an odds ratio of 2.4 (1.3, 4.5) and in age- and sex-adjusted logistic regression the odds ratio was unchanged. Combinations of NSAIDS and steroids were rare, and were prescribed in 1.7% of gout visits (1.1%, 2.8%). These proportions were similar in the 1993 to 2000 and 2001 to 2009 periods: 1.3% (0.6%, 2.8%) and 1.9% (1.1%, 3.5%) respectively.

## Discussion

**Figure 6:**
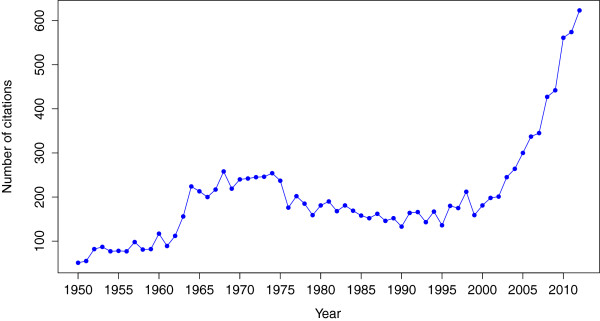
**Time trends in the number of publications in PubMed that mentioned gout in the title, abstract or Medical Subject Headings.** Data source: Alexandru-Dan Corlan. Medline trend: automated yearly statistics of PubMed results for any query, 2004. Web resource at URL: http://dan.corlan.net/medline-trend.html. Accessed 14 February 2012, Archived by WebCite at http://www.webcitation.org/65RkD48SV.

The number of ambulatory care visits for gout has increased substantially over time. Increase in age and changes in gender distribution in the survey participants explained some, but not all of the increases in the prevalence of gout. The large magnitude of this increase also cannot be explained by the modest changes in population incidence and prevalence of gout [2,14,15]. The NAMCS and NHAMCS data collection strategies have not changed substantially either. We propose that this phenomenon may be causally linked to the extensive patient and physician gout awareness programs led by manufacturers of urate-lowering therapies and entities such as the Gout and Uric acid Society since 2005. The date of 22 May was designated as the *Gout Awareness Day*. Effective electronic advertisements were placed. As an example, one such advertisement campaign used a news feed placed in rotation on the News section of the *USA Today* website [16]. The advertisement attracted more than 1% click-through rate on the news feed and readers spent more than 2 minutes and 30 seconds with the content. In just two weeks and over 2 million clicks, more than 20,000 *USA Today* readers spent time learning about gout. The media attention to vice president Dick Cheney’s gout may have a role as well, just as the first lady, Betty Ford’s breast cancer diagnosis that was credited with a transient increase of breast cancer incidence in the mid 1970s [17]. Lastly, the number of publications on gout also showed a significant increase over time, in part due to the funding from manufacturers and in part from the interest generated from the arrival of new products to treat gout (Figure 6). While these possibilities might be interesting, they are not testable hypotheses in our datasets.

We considered other possible explanations for a steep increase such as the epidemic of obesity, increasing utilization of low-dose aspirin, and the higher utilization of high-fructose corn syrup or such dietary changes, but rejected them as the rate of increase was so obviously disproportionate to such long-term trends.

To our knowledge, this is the first nationally representative study that reports time trends in the use of gout medications in the US population. Table 2 summarizes all the recent studies that have addressed the epidemiology of gout and gout medications in the ambulatory care settings. The designs, scope of enrollment and study duration are too heterogeneous to permit head to head comparison with our study. The proportion of patients/visits with allopurinol prescriptions varies widely depending on the case definition used to identify gout. The proportion of visits of patients on colchicine seems to be comparable to the average proportion observed in the present study.

**Table 2:** Comparison of pharmacoepidemiology surveys of gout in ambulatory care settings

**Author (year), country**	**Data source**	**Hospital-based clinics included?**	**Specialist visits included?**	**Calendar years of observation**	**Case definition of gout**	**Number of gout cases/visits/episodes**	**Proportion of patients (%)**^**a**^			
							**Allopurinol**	**Colchicine**	**Any NSAID**	**Systemic steroids**
Primatesta (2011), US	1	No	Yes	1996 to 2008	ICD codes and gout medication prescription	177,637	36	18	43	20
Reidel (2010), US	1	No	Yes	1997 to 1998	ICD codes and gout medication prescription	9,595	66	15	NA	NA
Roddy (2010), UK	2	No	No	2001 to 2004	Proprietary diagnosis odes	673	23	15	73	0
Annemans (2008), UK	2	No	No	2000 to 2005	ICD codes and/or medical record text mentions of gout, and prescription	34,071	89	16	89	26
Annemans (2008), UK	2	No	No	2000 to 2005	ICD codes and/or medical record text mentions of gout, and prescription	34,797	93	15	80	14
Harris (1995), UK	3	No	No	1993	General practitioner diagnosis	2,865	46	NA	NA	NA
Mikuls (2005), UK	2	No	No	1990 to 1999	Proprietary diagnosis codes	63,105	25 to 30	1 to 3	40 to 66	5%
Cea Soriano (2011), UK	2	No	No	2000 to 2007	Proprietary diagnosis codes	24,768	28	14	19	NA
Krishnan (2005), US	4	No	Yes	2002	ICD codes and prescriptions	206^a^	69	4.6	18	6.9
Present study, US^c^	4	Yes	Yes	1993 to 2009	ICD codes alone	100.4 million (1,634^b^)	35	15	34	10
Present study, US^c^	4	Yes	Yes	1993 to 2009	ICD codes and/or prescriptions	35.9 million (4,781^b^)	69	17	23	6.8

Settings type 1) Medical and pharmacy insurance claims; 2) General Practice case records with linked pharmacy records; 3) Survey of General practitioners; 4) Nationally representative sample survey of all ambulatory care visits including hospital clinics. ^a^Weighted proportions; ^b^Unweighted number of observations in the dataset; ^c^Cumulative, over the study period. NSAID, non-steroidal anti-inflammatory drugs; NA, not available.

The trends that we have documented in urate-lowering medication are similar to those observed in the UK ambulatory-care settings with stable use of all medications except anti-inflammatory drugs, which showed a significant decline [18]. Ever since the introduction of allopurinol as a therapy by Gertrude Elion, allopurinol had held sway as the mainstay of urate-lowering therapy for gout [15,19]. Nevertheless, allopurinol has been under-prescribed and under-dosed, resulting in poor urate control and consequently worse health outcomes and higher costs of care [5,10,20-27]. Unlike the UK data where the proportion of patients with urate-lowering therapies was between 25% and 30%, the proportion of prescriptions for allopurinol in our study was much higher.

Recently, uricosuric therapy has generated much interest on account of availability of newer agents [28-30]. Probenecid, an old uricosuric agent, gained popularity transiently in the past, but has fallen out of favor due to the dosing schedule, concerns about urolithiasis and reduced efficacy in the presence of azotemia [29]. In the present study probenecid prescriptions were observed to increase over time, but these increases were too few to reach statistical significance.

The combination of xanthine oxidase inhibitors and uricosuric agents as a means to achieve better urate efficacy did not gain popularity in light of earlier pharmacokinetic studies suggesting that probenecid increases renal clearance of oxypurinol, the active metabolite of allopurinol [31]. The number of prescriptions for combination therapy of probenecid and allopurinol was negligible, although there are no explicit adverse effects associated with combination therapy, even though more recent clinical studies suggest that the hypouricemic effects of these two drugs may be additive [32]. With increasing acceptance of the *treat-to-target* approach, we would expect a greater role for the combination therapy with xanthine oxidase inhibitors and uricosuric agents.

Gout in the general practice settings is often an episodic disease; the treatment is also often ad hoc, using colchicine and anti-inflammatory drugs. Anti-inflammatory drugs, often recommended as a first line of therapy, have been falling out of favor due to the recognized adverse effects such as gastrointestinal bleeds, renal failure and hypertension [33,34]. The overall utilization of anti-inflammatory drugs has decreased in the US. Colchicine, an ancient remedy for acute gout flares, has been utilized more frequently. It is unlikely that any of the colchicine prescriptions recorded during the latest year of the study, 2009, was for the proprietary formulation. Systemic steroids offer inflammation control for acute gout flares and may be preferable for those with renal disease and other contraindications for colchicine and anti-inflammatory drugs. The utilization of these did not change over time.

The estimates presented here need to be considered in the context of the characteristics of the data collection strategies. First, these data do not include those from federal hospitals such as the Veterans Affairs-owned hospitals. Many such hospitals are known to have a high prevalence of gout. The uninsured patients are likely to be under-represented in ambulatory-care data. Thus, our estimates are likely to be lower than the true number of visits. Second, although the diagnosis of gout was made by the physician, it was not standardized, leaving room for misclassification errors. Third, the denominator of our estimated proportions is the total number of visits, not total number of patients or total number of prevalent cases of gout. Fourth, not prescribing a specific medication during a visit does not necessarily represent a lack of knowledge, interest or intent on the part of the physician; it may reflect individual clinical realities (for example, contraindications, competing hazards from therapeutic alternatives) and patient preference [35-37]. Such granularity of data, which is needed to determine the appropriateness of prescription, is not collected in the Ambulatory care surveys.

Comparisons of the estimates in the present study with those from contemporaneous population surveys are not easy but are tempting. The NHANES 2009 to 2010 surveys estimated that there were about 8 million cases of gout prevalent in the US, whereas the present study estimated 12 million outpatient visits in 2009. However, the proportion of gout as a cause of all-cause ambulatory visits in the present study is smaller at 0.80% (0.75%, 0.85%) than the prevalence rate of gout estimated in the NHANES 2009 to 2010 (3.8%). These two comparisons suggest that the health care utilization rates for gout may be lower than what might be expected from the population prevalence of gout. The disparities have other potential explanations as well: in the present study, the data on diagnoses were restricted to active problems, with the premise that no more than three problems are likely to be addressed in a single ambulatory care visit. Thus inactive problems, such as remote history of gout, are unlikely to be recorded. In the NHANES, there was a dedicated question about physician/provider-diagnosed gout. The implication is that the estimates of gout from the present study may reflect the point prevalence as opposed to lifetime prevalence assessed by the NHANES. Second, the mode of diagnosis in the present study was physician diagnosis and physician-prescribed medications, whereas in NHANES it was health-care provider diagnosed gout. Some of the disparities may reflect higher diagnostic errors in the NHANES data than the present data.

## Conclusions

In conclusion, we have documented the ambulatory care utilization of gout and gout medications in the 1990s and the first decade of the millennium. These data would serve as a baseline for future pharmaco-epidemiologic studies that will include the newer therapies and help identify areas in health care delivery where quality of care can be optimized and outcomes improved.

## Abbreviations

ICD9: International Classification of Diseases 9^th^ revision; NAMCS: National Ambulatory Medical Care Surveys; NHAMCS: National Hospital Ambulatory Medical Care Surveys; NHANES: National Health and Nutrition Examination Survey; NSAID: Nonsteroidal anti-inflammatory drug; PSU: Primary sampling units.

## Competing interests

The work presented in this manuscript was funded in part by an investigator-initiated grant from URL Pharma (presently Takeda Pharmaceuticals). In compliance with the agreement with Stanford University, the sponsors did not have any role in the design, execution, data analysis, interpretation or drafting of this manuscript. These data are available in the public domain and statistical codes used for the present analyses are available from the authors.

## Authors’ contributions

EK conceived the study, accessed the data, designed the analysis, interpreted the data and wrote the first draft. LC co-designed the analysis, analyzed the data, assisted in interpretation of the data and participated in the writing and editing of the manuscript. Both authors read and approved the final version of the manuscript.
